# Case report: A large gastric calcifying fibrous tumor treated with endoscopic submucosal excavation

**DOI:** 10.3389/fonc.2024.1385695

**Published:** 2024-08-12

**Authors:** Ziyou Zhong, Zhenguo Li, Yufeng Xing, Shaoju Guo

**Affiliations:** ^1^ The Fourth Clinical Medical College, Guangzhou University of Chinese Medicine, Shenzhen, Guangdong, China; ^2^ Department of Gastroenterology, Shenzhen Traditional Chinese Medicine Hospital, Shenzhen, Guangdong, China; ^3^ Department of Pathology, Shenzhen Traditional Chinese Medicine Hospital, Shenzhen, Guangdong, China; ^4^ Department of Liver Diseases, Shenzhen Traditional Chinese Medicine Hospital, Shenzhen, Guangdong, China

**Keywords:** calcifying fibrous tumor, endoscopic submucosal excavation, stomach, submucosal tumor, case report

## Abstract

Gastric calcifying fibrous tumor (CFT) is a rare benign mesenchymal tumor. Several previous studies have reported surgical resection for gastric CFT larger than 20mm for the difficulty in preoperative diagnosis. Here, we report a rare case of large gastric CFT treated with endoscopic submucosal excavation (ESE). A 70-year-old woman presented with recurrent epigastric pain and underwent endoscopy, which revealed a 35mm-sized submucosal tumor in the gastric body. ESE was performed after imaging examination and endoscopic ultrasonography. En bloc resection was achieved, but due to the specimen’s substantial size and difficulty in mincing, it posed challenges for removal through the mouth. Finally, the specimen was temporarily placed in the stomach and was completely removed two days later. The diagnosis was confirmed based on pathological and immunohistochemical findings. There was no recurrence during the patient’s 11-month follow-up. We provided a case report related to the diagnosis and endoscopic treatment for large gastric CFT. In addition, our experience of temporarily leaving a large postoperative specimen, considered a benign lesion, in the stomach for later removal was successful but requires appropriate timing to avoid blockage of the gastrointestinal tract.

## 1 Introduction

Calcifying fibrous tumor (CFT) is a rare benign mesenchymal tumor, remaining unclear etiology and pathogenesis. The tumor was first described as a fibrous tumor with psammomatous calcifications and lymphoplasmacytic cell infiltration in the deep soft tissue in two children by Rosenthal and Abdul-Karim in 1988 (1). In 2002, Nascimento et al (2) reported local recurrence occurred in 3 patients with calcifying fibrous pseudotumor, and the World Health Organization recommended its current name (3). The lesion can occur in various sites, and the common locations are the stomach, small intestine, pleura, neck, mesentery, mediastinum, and peritoneum (4). A review of CFT in the gastrointestinal tract has concluded that distinguishing gastric CFT from gastrointestinal mesenchymal tumors, particularly gastrointestinal stromal tumors, is challenging due to the absence of typical clinical and imaging findings (5). Definitive diagnosis is made by histology, which is characterized by a spindle cell proliferation with an abundant hyalinized stroma, along with focal dystrophic calcifications and a lymphoplasmacytic infiltrate (5, 6). Therefore, gastric CFT is often missed in diagnosis or misdiagnosed as gastrointestinal stromal tumor (GIST) before operation (7–9). Up to date, surgical resection is the primary treatment for CFT (10). Although a few previous studies have reported endoscopic treatment for gastric CFT with a tumor diameter of less than 20mm, surgical excision is the preferred approach for larger ones (7, 11).

Here, we present a case of large gastric CFT treated with endoscopic submucosal excavation (ESE), aiming to raise awareness of this rare condition. In addition, we share our experience in dealing with a huge postoperative specimen that was hard to remove through the mouth due to difficulty in mincing.

## 2 Case description

**Figure 1:**
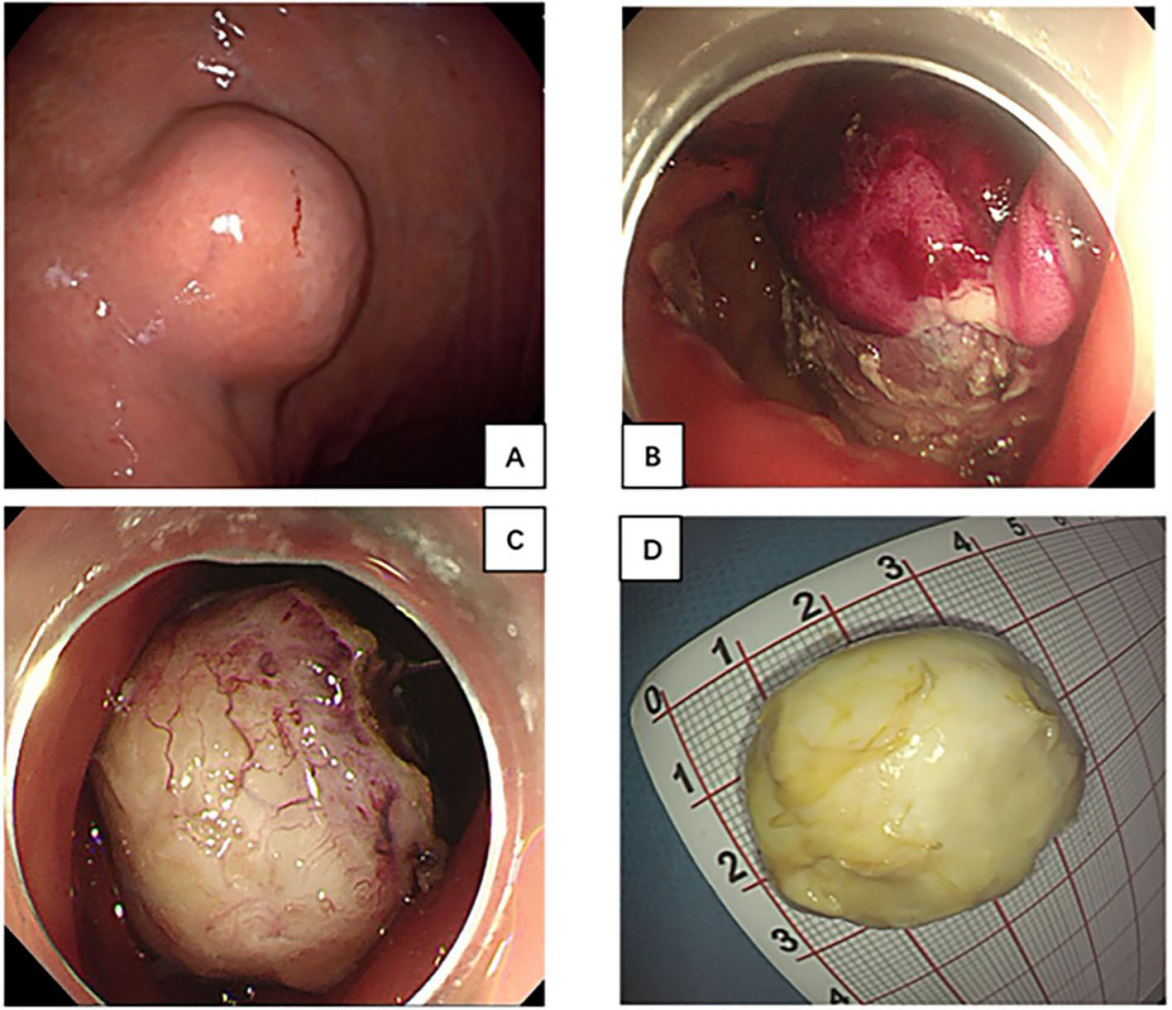
**(A)** Gastroscopy showed a large submucosal tumor in gastric body; **(B, C)** The lesion treated with endoscopic submucosal excavation and En bloc resection was achieved. **(D)** Specimen was removed through the mouth 2 days later.

**Figure 2:**
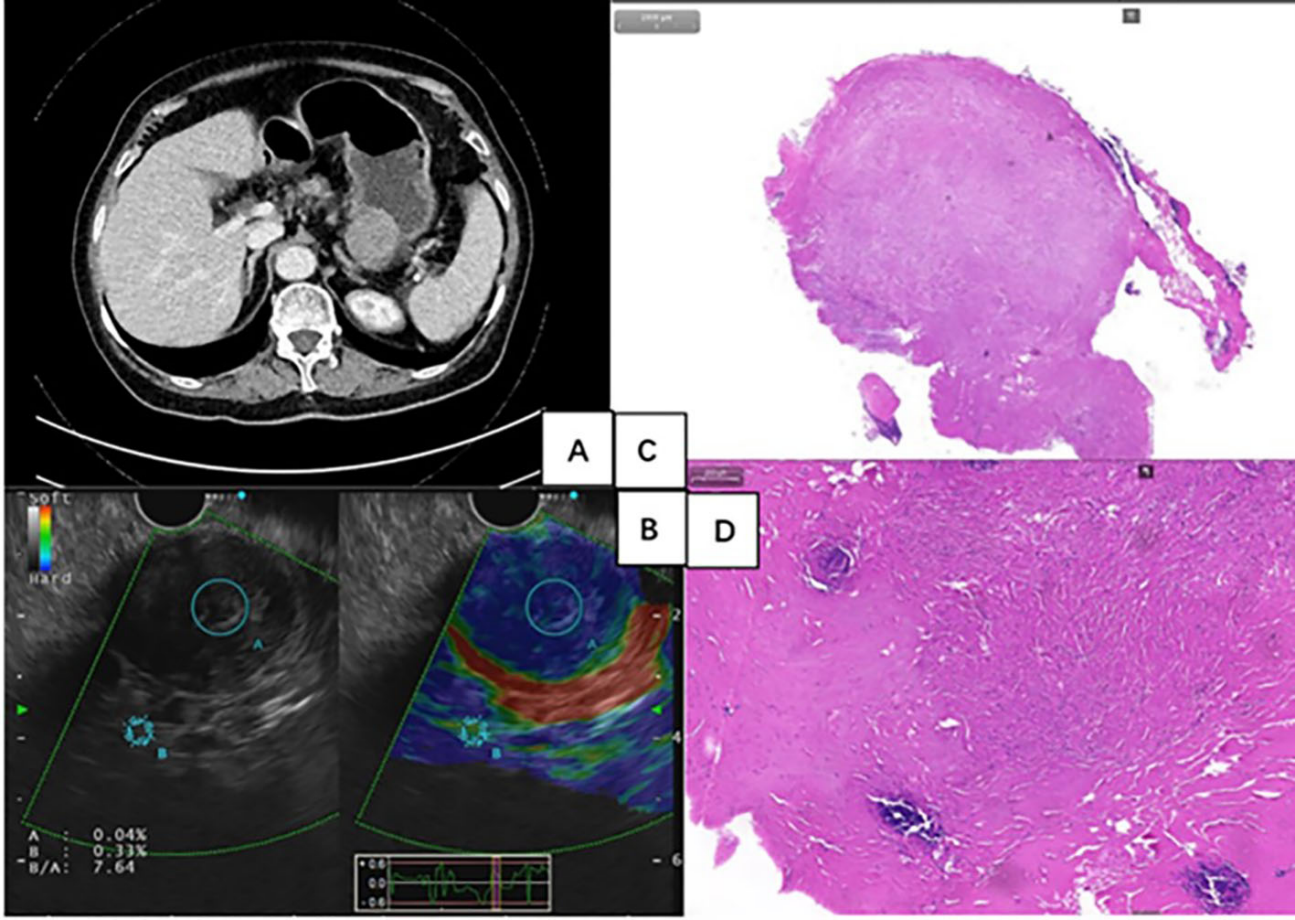
**(A)** Contrast-enhanced computed tomography scan revealed a 37mm×37mm×30mm-sized, well-defined, mild-to-moderate enhanced, and homogeneous round mass. **(B)** Endoscopic ultrasonography showed a hard, heterogeneous hypoechoic tumor, appearing to originate from muscularis propria. **(C, D)** Histology showed the presence of abundant hyalinized collagen, along with focal dystrophic calcifications and a lymphoplasmacytic infiltrate (Hematoxylin-eosin stain).

A 70-year-old woman with recurrent epigastric distension and pain for 5 years presented to our hospital. She reported no symptoms of acid reflux, heartburn, nausea, vomiting, diarrhea, melena, or unintentional weight loss. Additionally, her past medical history and family history were non-specific. The routine physical examination was unremarkable. Then, the patient received a gastroscopy and C13 breath test. The result showed a large submucosal tumor (SMT) in the gastric body (**Figure 1A**) and Helicobacter pylori (H. pylori) infection. Therefore, the outpatient physician has recommended hospitalization for further diagnosis and treatment of gastric SMT and eradication treatment for H. pylori. The patient received quadruple therapy to eradicate H. pylori, which included ilaprazole enteric-coated tablets 5mg twice daily, compound bismuth aluminate granules 2 sachets twice daily (Each sachet contains 200mg of bismuth aluminate.), doxycycline hydrochloride tablets 0.1g twice daily, and amoxicillin capsules 1g twice daily. Subsequently, the patient was admitted to the Department of Gastroenterology for further examination and treatment. The laboratory testing was notable for a tumor mark carbohydrate antigen 72-4 level of 10.4 U/ml (reference, 0-6), while routine blood, liver, and kidney function, electrolytes, myocardial enzymes, and other tumor marks showed no abnormalities. Contrast-enhanced computed tomography (CT) scan of the chest, entire abdomen, and pelvis revealed a 37mm×37mm×30mm-sized, well-defined, mild-to-moderate enhanced, and homogeneous round mass in the stomach (**Figure 2A**). The CT scan also indicated a value of around 43 Hounsfield Units (HU), with no apparent liquefaction necrosis, clear fat space surrounding the area, and no observable enlargement of lymph nodes. Endoscopic ultrasonography (EUS) showed the heterogeneous hypoechoic tumor with an elasticity score of 7.64, which appeared to originate from muscularis propria (**Figure 2B**). EUS-guided fine needle aspiration (EUS-FNA) was performed. The cytologic examination showed scattered spindle cells and was inconclusive for diagnosis.

**Figure 3:**
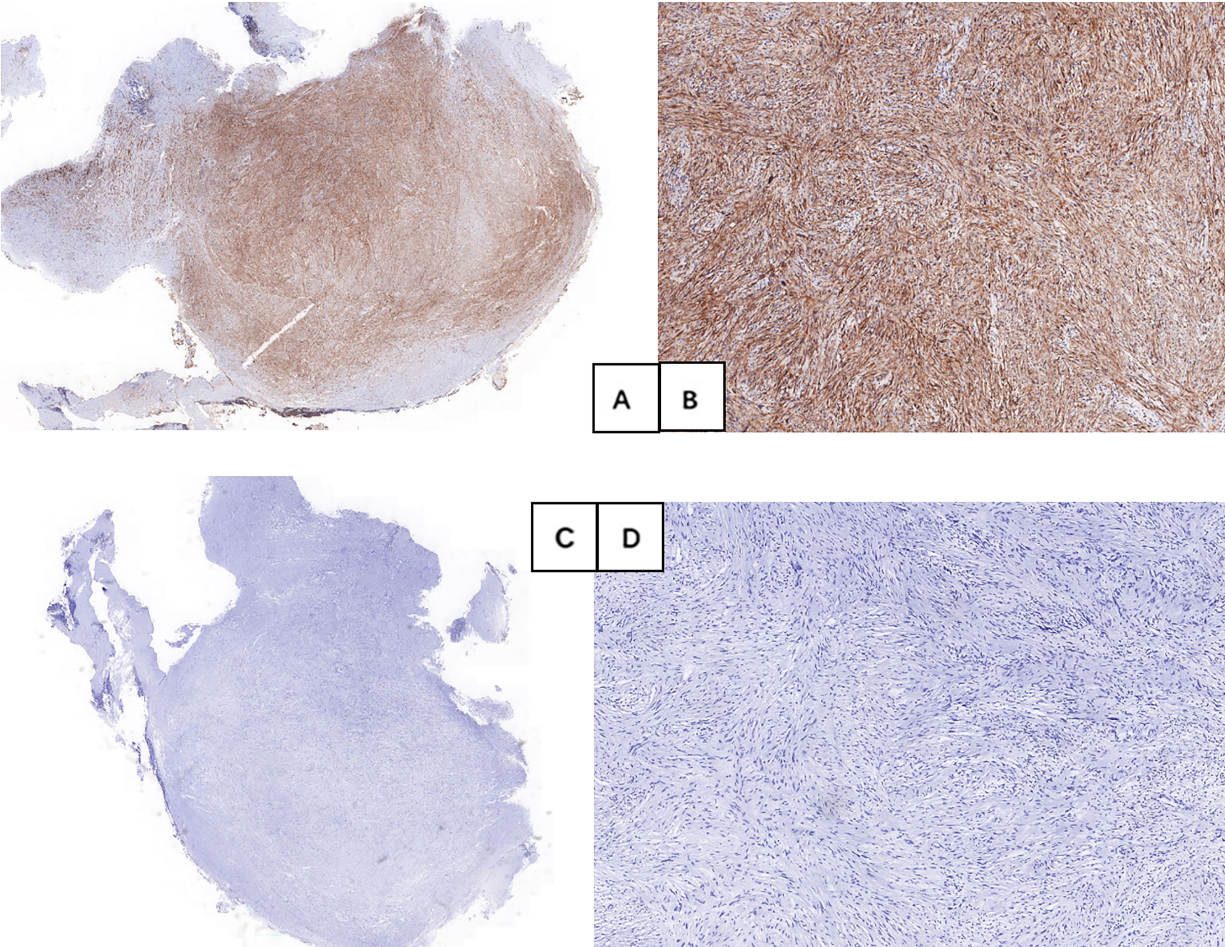
The Immunohistochemical testing was positive for vimentin **(A, B)**; and negative for FactorXIIIa, CD117, CD34, DOG-1, SMA, Desmin, S100, SDHB, FH, Caldesmon, Syn, CgA, GFAP, ALK and Bcl-2 **(C, D)**.

Surgery was initially recommended as the optimal course of treatment, but the patient adamantly declined surgery for her advanced age. As a result, an ESE procedure was performed at the request of the patient and her family. En-bloc resection was achieved without any significant adverse events (**Figures 1B, C**). However, the specimen’s substantial size and difficulty in mincing posed challenges for removal through the mouth. With the patient’s family’s full informed consent, the specimen was temporarily placed in the stomach and was completely removed using a retrieval net two days later (**Figure 1D**). During the 2 days when the specimen remained in the patient’s stomach, she was instructed to abstain from food, bed rest, and reduce activity to minimize gastrointestinal motility. Regarding treatment within 48 hours post-surgery, routine measures included administering prophylactic antibiotics, and proton pump inhibitors to suppress acid secretion, as well as providing fluid and nutritional support, without any additional special measures. Histology diagnosed gastric CFT with the presence of abundant hyalinized collagen, along with focal dystrophic calcifications and a lymphoplasmacytic infiltrate (**Figures 2C, D**). Immunohistochemical testing was positive for vimentin and negative for FactorXIIIa, CD117, CD34, DOG-1, SMA, Desmin, S100, SDHB, FH, Caldesmon, Syn, CgA, GFAP, ALK and Bcl-2 (**Figure 3**). The Ki-67 level was approximately 3% positive. Additionally, special staining revealed negativity for elastic fibers and positivity for mesh staining. Postoperative recovery was uneventful. The patient received regular follow-up at the clinic and no recurrence was observed with re-gastroscopy for 4 months after tumor excision. However, the patient did continue to experience mild intermittent epigastric distension during the 11-month follow-up period. In particular, the patient returned to the outpatient clinic for a follow-up visit and received traditional Chinese medicine treatment for intermittent epigastric distension that occurred 6 months after surgery.

## 3 Discussion

Gastric CFT is a benign lesion that was considered rare in the past, but with the popularization of endoscopy, perhaps its incidence is not as rare as we think, although its incidence has not been reported (5). Its definitive diagnosis is made by histology, which is characterized by a spindle cell proliferation with an abundant hyalinized stroma, inflammation, and interspersed calcifications. Immunohistochemically, the spindle cells stain positively for vimentin, show variable expression of CD34 and Factor XIIIa, and are negative for CD117, DOG-1, SMA, Desmin, ALK-1, and S100 (5, 6, 12, 13). These immunohistochemical staining results are used to differentiate it from other common spindle cell lesions. For example, inflammatory myofibroblastic tumors are always positive for SMA and ALK-1, leiomyomas are always positive for Desmin and Caldesmon, GIST is positive for CD117, CD34, and DOG-1, schwannomas are positive for S100, etc. (5). Accordingly, we conducted comprehensive immunostaining in this case to exclude other pathologies. As such, it is crucial to fully comprehend its clinical, endoscopic, and imaging characteristics before surgical intervention, as this understanding plays a critical role in determining the subsequent treatment plan. Most gastric CFTs have been the subject of case reports (7, 11, 14, 15). For the clinical feature, the tumor appears to have no sex predilection and occurs more often in middle-aged people (5, 10, 12). They are typically asymptomatic and discovered incidentally (4). If symptoms are present, they are usually non-specific and commonly include abdominal pain and discomfort (6). In gastroscopy, gastric CFT manifests as an SMT with a round or oval shape, and most tumors are in the body of the stomach (10, 14). The regular shape may be related to its benign biological behaviors.

As a noninvasive examination method, CT offers numerous irreplaceable advantages in the evaluation of gastric neoplasms. It not only clearly depicts the locations of lesions but also accurately assesses the relationships between these lesions and the surrounding tissues or organs. However, only a few CT images have been reported in the previous literature. Wang et al. analyzed six reports on CT findings of gastric tumors that small size (mean: 2.1 cm), a high unenhanced CT attenuation value (mean: 51.9 HU), and mild-to-moderate enhancement (mean: 23.1 HU) should facilitate diagnosis of gastric CFTs (16). Zhang et al. also reported a soft-tissue density mass with calcification and slight enhancement in CT images (17). In our case, CT shows a well-defined, mild-to-moderate enhanced, and homogeneous round mass, similar to those described in their findings.

Only rare reports of gastric CFTs contain well-documented EUS findings. The EUS features of the lesions were heterogeneous and well-defined, with most lesions appearing hypoechoic (10, 18, 19). Calcifications and postacoustic shadowing were also observed in some of the lesions (10, 19). Additionally, it has been observed that most of them originate in the muscularis propria, followed by the submucosal layer and serous layer (10, 19). Furthermore, ultrasound elastography in our case showed a hard texture of the lesion. To date, there have been only two reports on EUS-FNA of gastric CFTs. However, the results of cytologic examination in both cases were nondiagnostic (7, 20). Our case yielded similar results, suggesting that EUS-FNA may pose challenges in establishing a definitive diagnosis. There are several potential reasons for this difficulty. Firstly, the procedure often yields a relatively small amount of tissue, which may limit the diagnostic accuracy. Secondly, pathologists may lack familiarity with this particular disease, further hindering the interpretation of the obtained samples. A literature reported that contrast-enhanced CT is an accurate technique for discrimination of GIST and benign gastrointestinal mesenchymal tumors (21), which may also apply equally to CFTs but needs further evaluation. Additionally, when differentiating between malignancy and CFTs proves challenging, positron emission tomography-computed tomography (PET-CT) may offer a novel diagnostic option, although there are no literature reports currently.

At present, surgical resection, specifically wedge resection, is the primary treatment for CFT (5, 7, 8, 10, 15, 20, 22–24). However, advancements in endoscopic technologies have introduced endoscopic interventions as potential alternatives for removing gastric CFTs. Qiang et al. concluded that endoscopic treatments, especially endoscopic submucosal dissection (ESD)/endoscopic full-thickness resection (EFR), seem to be feasible and safe procedures for managing CFT with relatively few complications and low mortality through retrospectively analyzed total of 4 gastric CFTs treated with ESD or EFR (18). In addition, other two studies have reported that ESE also can be considered an alternative method for resecting gastric CFTs (11, 25). However, in all the aforementioned endoscopic cases, the size of the lesions was less than 2cm. This is mainly due to the challenge of distinguishing them from other SMTs, particularly stromal tumors, and the potential risk of malignancy associated with these lesions. A Chinese consensus on SMTs recommends that lesions with no or very low risk of lymph node metastasis, which can be completely resected by endoscopic techniques, with low risk of residual and recurrence are suitable for endoscopic resection if necessary (26). European Society of Gastrointestinal Endoscopy recommends EUS-guided fine-needle biopsy or mucosal incision-assisted biopsy equally for tissue diagnosis of SMTs ≥ 20 mm in size (27). In our case, the decision to perform ESE for the large lesion was made based on the benign nature of the lesion as determined by clinical performance, CT imaging, and EUS results, as well as the patient’s preferences. During the operation, we encountered an unexpected situation: the specimen was too large to be completely extracted orally as anticipated. Given the preoperative assessment indicating a benign condition, we opted to leave the specimen in the stomach for two days to allow for the digestion and dissolution of the soft tissue on the surface of the tumor before removal. It’s worth noting that for the 2 days when the specimen remained in the patient’s stomach, she was instructed to refrain from eating, rest in bed, and reduce activity to minimize gastrointestinal movement. In our experience, these measures may be necessary to prevent excessive gastrointestinal peristalsis, which could result in the specimen migrating downward from the stomach and causing gastrointestinal obstruction. Upon extraction, the measured size of the specimen was approximately 35mm×30mm. To our knowledge, this represents the first reported case of delayed retrieval of a massive specimen from the stomach. Indeed, endoscopic treatment of SMTs is an effective way not only for enhancing patients’ quality of life for patients but also for reducing the economic and emotional burden on patients’ families and society, while conserving national medical resources (26).

CFT is considered a distinctive benign mesenchymal neoplasm with a low risk of recurrence, approximately 20% (3 out of 15) in one study, but the recurrent lesions were all in soft tissue (2). To date, no recurrence has been reported in any of the cases with gastric CFTs that underwent local resection (10, 12). No deaths owing to CFT were reported in the literature and as a result, long-term survival was estimated at a rate of 100% (4). Many professionals have suggested that asymptomatic gastrointestinal SMTs (< 30 mm) could be followed up with periodic endoscopy. Therefore, we recommend that patients with preoperative evaluation of suspected CFT can be followed up and monitored. Endoscopic treatment is preferred if the patient with tumors smaller than 3.5cm has symptoms or expresses a desire for treatment, while surgical resection may be more appropriate for a tumor larger than 3.5cm because the lesion is too large to be removed completely through the mouth.

In summary, we systematically described the clinical, endoscopic, imaging features and endoscopic treatment for a rare large gastric CFT. Our experience of temporarily leaving a large postoperative specimen, considered a benign lesion, in the stomach for later removal was successful but requires appropriate timing to avoid blockage of the gastrointestinal tract. However, there are limitations in that this is a case report of a singular patient and a relatively short follow-up period, so larger cohort studies and longer follow-up times are needed to define the ideal diagnosis and treatment for gastric CFT.

## Data Availability

The original contributions presented in the study are included in the article/**Supplementary Material**. Further inquiries can be directed to the corresponding author.
