# Supplementary material for: Case report: A large gastric calcifying fibrous tumor treated with endoscopic submucosal excavation
Source: Front Oncol. 2024 Aug 12;14:1385695. doi: 10.3389/fonc.2024.1385695 (PMC11345178; doi:10.3389/fonc.2024.1385695)
Supplement: Supplementary file 1 [file DataSheet1.pdf]

Supplementary Figures

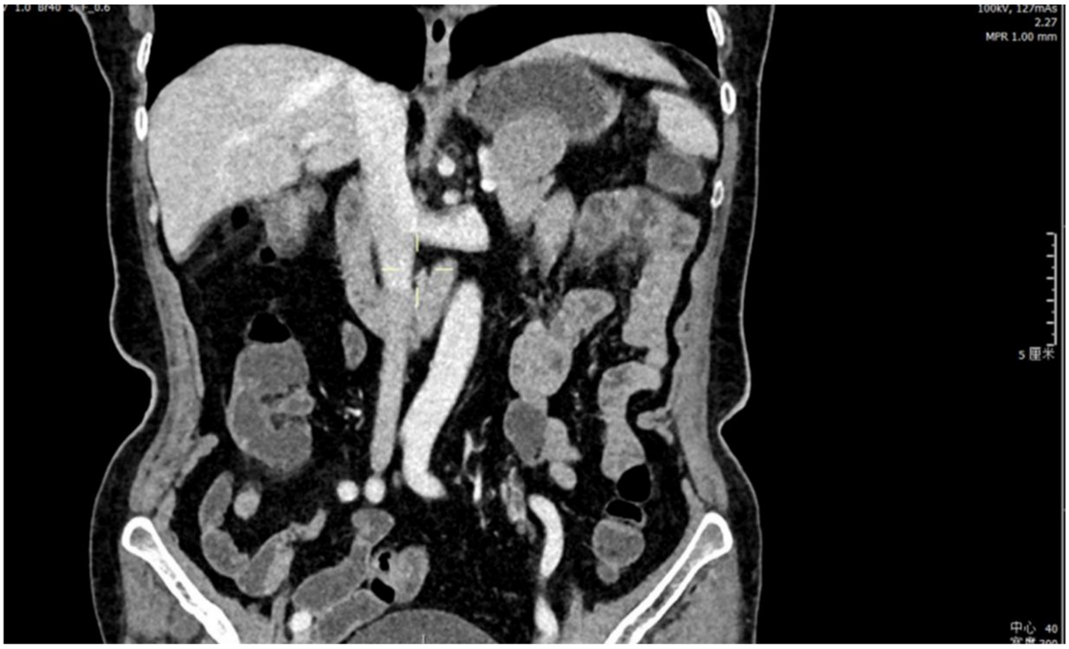

Figure 1. Coronal contrast-enhanced computed tomography scan revealed a well-defined, mild-to-moderate enhanced, and homogeneous round mass.

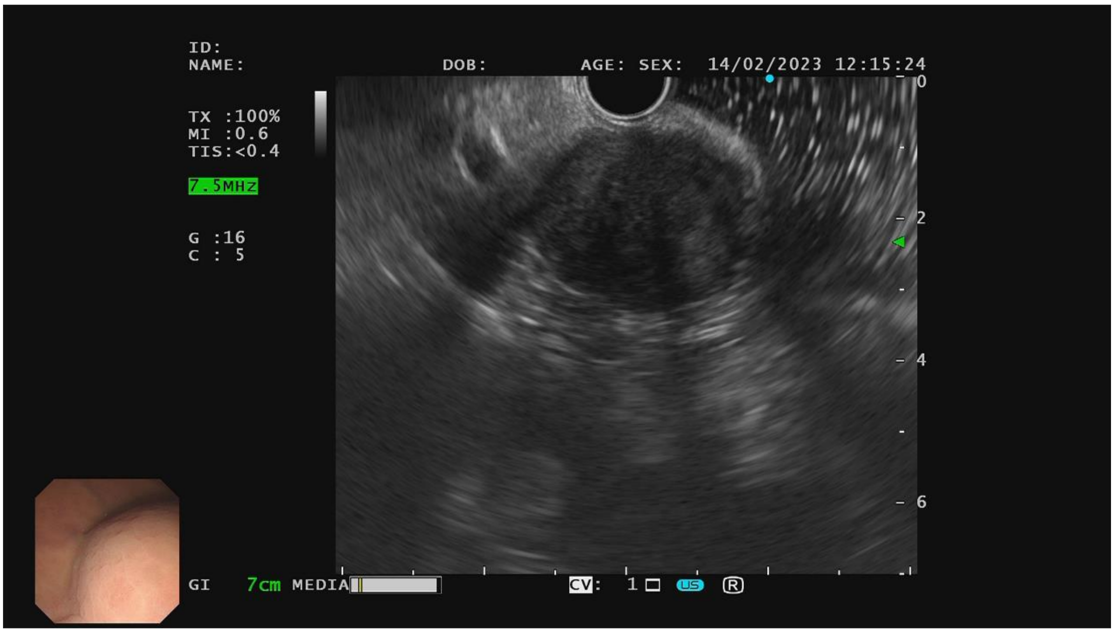

Figure 2. Endoscopic ultrasonography showed a heterogeneous hypoechoic tumor, appearing to originate from muscularis propria.

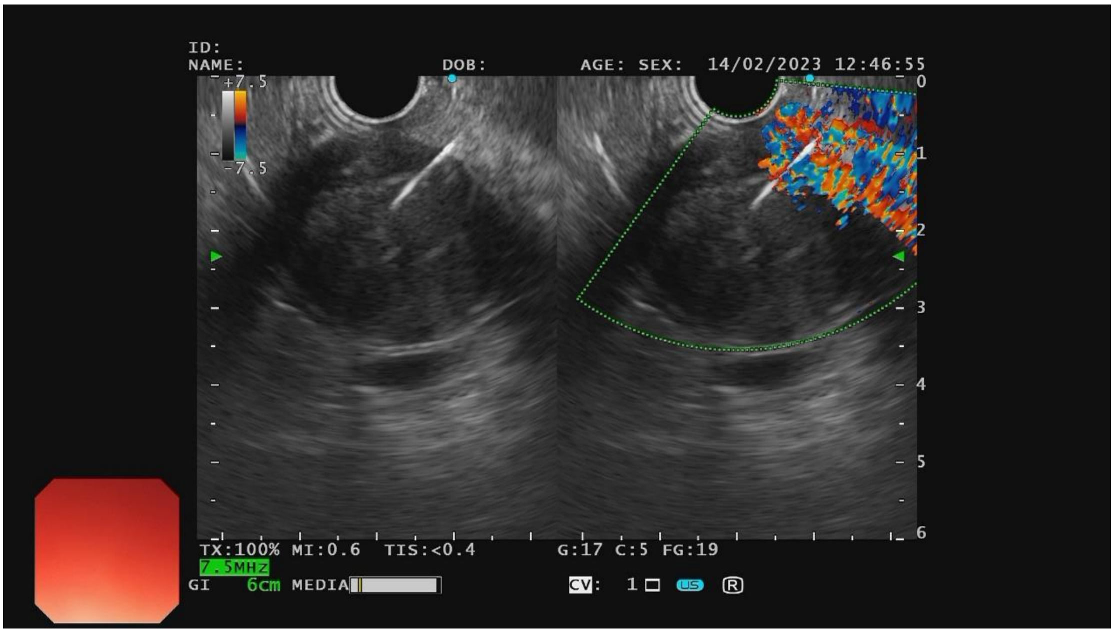

Figure 3. Performed EUS-guided fine needle aspiration.

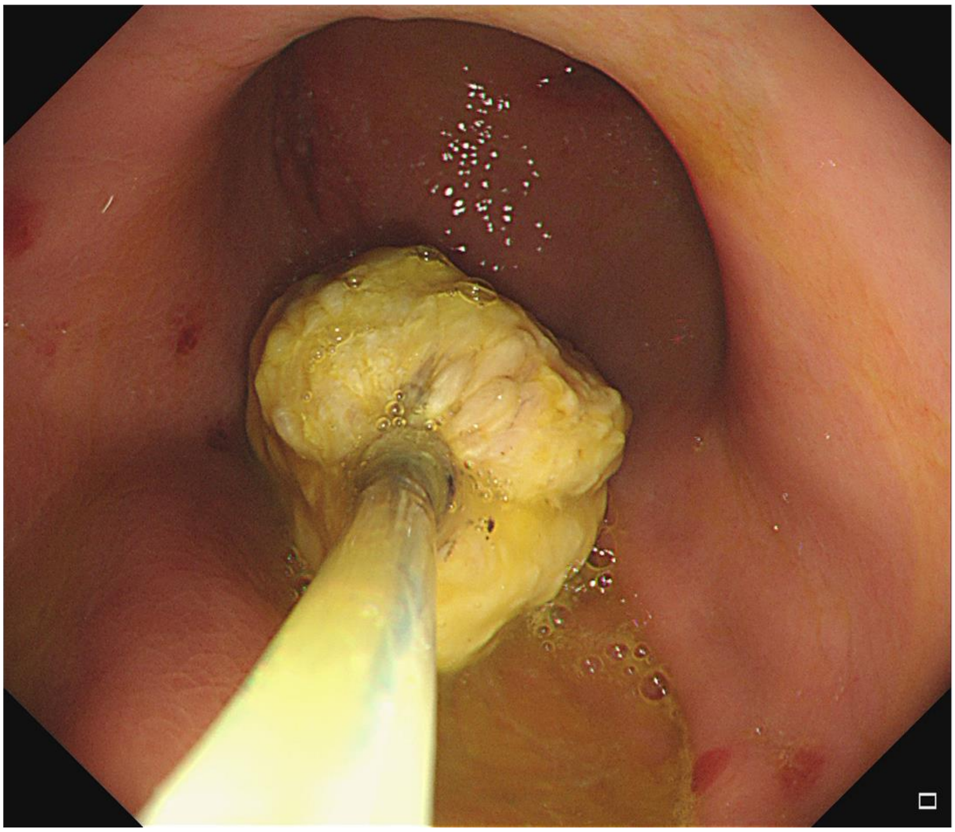

Figure 4. Specimen was removed using a retrieval net 2 days after endoscopic operation.

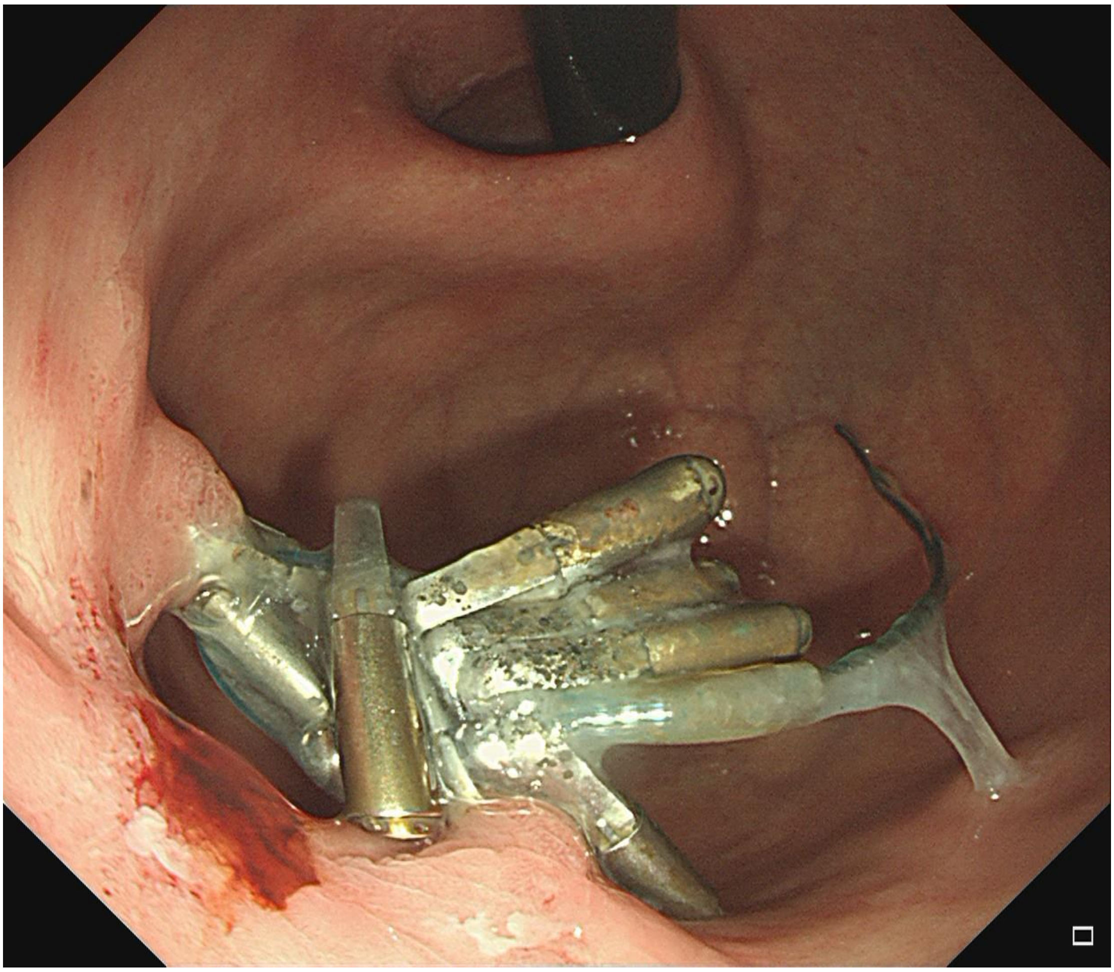

Figure 5. Re-gastroscopy revealed healing scar tissue and residual metal clips 4 months after tumor excision.
